# Novel *NFIX* variant in a patient with Malan syndrome and associated Chiari type I malformation: a case report

**DOI:** 10.3389/fped.2026.1794137

**Published:** 2026-04-10

**Authors:** Michele Minerva, Anna Maria Pinto, Filippo Toninelli, Andrea Francioni, Lorenzo Perilli, Elisa Laschi, Luisa Lonoce, Federica Lotti, Maria Rosaria Curcio, Chiara Fallerini, Alessandra Renieri, Salvatore Grosso

**Affiliations:** 1 Clinical Pediatrics, Department of Molecular Medicine and Development, University of Siena, Azienda Ospedaliero-Universitaria Senese, Siena, Italy; 2 Department of Biotechnology, Chemistry and Pharmacy, University of Siena, Siena, Italy; 3 Medical Genetics, Azienda Ospedaliera Universitaria Senese, Siena, Italy; 4 Department of Neurosciences, Rehabilitation, Ophthalmology, Genetics, Maternal and Child Health, University of Genoa, Genoa, Italy; 5 Department of Neuromuscular Diseases, UCL Queen Square Institute of Neurology, London, United Kingdom; 6 Medical Genetics, University of Siena, Siena, Italy; 7 Med Biotech Hub and Competence Centre, Department of Medical Biotechnologies, University of Siena, Siena, Italy

**Keywords:** Chiari malformation, macrocephaly, Malan syndrome, *NFIX*, overgrowth

## Abstract

**Background/objectives:**

Malan syndrome (MALNS) is a rare overgrowth disorder caused by pathogenic Nuclear Factor I × (*NFIX*) gene variants, and characterized by postnatal overgrowth, macrocephaly, developmental delay, intellectual disability and distinctive facial features. Chiari type I malformation (CMI), a condition where the cerebellar tonsils extend below the foramen magnum, has been observed in some patients with MALNS, although the exact relationship between these disorders remains unclear. The objective of this case report is to describe a novel *NFIX* variant in a patient with MALNS and associated CMI. This case adds to the literature on *NFIX* variants in patients with CMI and underscores the potential benefit of early genetic testing for diagnosis and management.

**Case presentation:**

We describe a patient with clinical features consistent with MALNS, including macrocephaly, developmental delay, and typical craniofacial features. Brain Magnetic Resonance Imaging (MRI) revealed the presence of CMI. Genetic testing identified a novel heterozygous variant in *NFIX*, not previously described in the literature.

**Conclusion:**

This case contributes to the clinical and molecular characterization of MALNS by linking a previously unreported *NFIX* variant to CMI. The case underlines the importance to take into account MALNS in patients presenting with overgrowth and CMI. Furthermore, we report a novel variant to improve diagnostic accuracy and genotype-phenotype correlation. Indeed, timely molecular diagnosis is essential to differentiate among overgrowth syndromes and to establish appropriate long-term clinical follow-up.

## Introduction

Overgrowth syndromes represent a wide group of clinically and genetically defined conditions which have been traditionally characterized by an increase of anthropometric features e.g., height, weight, and/or occipitofrontal circumference (OFC), usually above three standard deviations (or the 99th percentile) on growth charts compared to age and sex-matched peers (1).

Overgrowth can be generalized when it affects multiple organs and tissues, segmental when excessive growth is patchy or localized to specific organs. Generalized overgrowth typically presents as tall stature, macrocephaly, and/or abdominal organomegaly, and it is commonly caused by germline genetic variants. These disorders are frequently associated with other phenotypic abnormalities, including developmental delay, intellectual disability and an increased risk of cancer (2).

Among the overgrowth syndromes, Sotos syndrome (SS) is one of the most extensively studied and clinically recognized. SS is caused by haploinsufficiency of the Nuclear receptor Set Domain containing protein 1 gene (*NSD1*) and is characterized by learning difficulties, variable medical issues - such as cardiac and renal anomalies, and seizures - as well as a distinctive facial gestalt. Typical craniofacial features include dolichocephaly with a broad forehead, sparse frontotemporal hair, a prominent chin, and downslanting palpebral fissures (3).

A landmark study by Malan et al. (2010) identified *NFIX* haploinsufficiency as the cause of a novel overgrowth syndrome clinically similar to SS, later recognized as MALNS. The authors demonstrated that the nature of the *NFIX* mutation - and its interaction with nonsense-mediated mRNA decay (NMD) - dictates the resulting phenotype. Loss-of-function (LoF) mutations subject to NMD lead to the milder MALNS, characterized by postnatal overgrowth, macrocephaly, intellectual disability, and a recognizable facial gestalt. In contrast, mutations that escape NMD produce truncated NFIX proteins with dominant-negative effects, resulting in the more severe Marshall–Smith syndrome, associated with accelerated bone maturation, respiratory complications and early mortality. This study highlighted the importance of allele-specific effects in *NFIX*-related disorders, providing a mechanistic basis for their clinical heterogeneity (4).

Since its initial description in 2010 (4), MALNS remains an ultra-rare disorder, with an estimated prevalence of approximately 1:1,000,000, and an expanding phenotypic spectrum as additional cases are described (6, 7). Subsequently, additional cases consistent with MALNS were reported, allowing for the definition of its key clinical features. MALNS, previously known as Sotos syndrome type 2, is a genetic overgrowth condition marked by excessive growth, macrocephaly, distinctive facial features, cognitive impairment, and behavioral difficulties (5). The syndrome results from haploinsufficiency of the *NFIX* gene, typically due to heterozygous chromosomal microdeletions affecting the 19p13.2 region, or from LoF variants within the *NFIX* gene itself, most of which are located in exons 2 through 4 (6).

Missense mutations in *NFIX* have also been described in MALNS with a clustering of *NFIX* missense variants (at positions Ser106, Lys 113, Arg115, Arg116, Arg121, Lys125, and Arg128). Notably, these variants localize within the N-terminal DNA-binding/dimerization domain of *NFIX*, a functionally important region involved in DNA binding and transcriptional regulation. Many of the affected residues are positively charged amino acids, suggesting that alterations in this domain may disrupt protein–DNA interactions and contribute to the pathogenic mechanism (5). It has been suggested that NFIX proteins carrying missense variants may exhibit impaired nuclear localization or altered DNA-binding capacity. These alterations may result in reduced *NFIX* transcriptional activity and are compatible with a loss-of-function effect, although the precise pathogenic mechanisms of missense variants remain to be fully elucidated.

Among the associated clinical features, CMI has been occasionally described. However, the identification of novel *NFIX* variants remains crucial for refining the genotype-phenotype correlation.

In this report, we describe a patient with MALNS presenting with CMI and harboring a previously unreported pathogenic variant in *NFIX*. By sharing this case, we aim to contribute to the characterization of the clinical and molecular spectrum of the syndrome, and to support ongoing efforts to better understand the variability of its neurological manifestations.

## Case description

We describe the case of a second-born male child of non-consanguineous parents, without significant family history. The child was born at 39 weeks of gestation after a spontaneous normal pregnancy, via cesarean section for breech presentation. APGAR score and perinatality were reported to be normal. At birth, the patient presented with the following auxological parameters: weight 3620 g (72nd percentile), length 52 cm (84th percentile), and OFC 35 cm (61st percentile), according to Bertino et al. Neonatal Anthropometric Charts. At 7 months of age, OFC measured 48.5 cm (>97th percentile), based on WHO Anthropometric Charts.

Height and weight growth in the first years of life was reported as regular, with OFC growth consistently above the 97th percentile.

The cerebral ultrasound at 8 months of life was substantially normal, reporting only a slight enlargement of the frontal horns of the lateral ventricles and a large third ventricle, with a normal fourth ventricle and remaining cerebrospinal fluid spaces.

The child showed a delay in neuromotor development, reaching an independent sitting position at 10 months, walking independently at 18 months, and speaking his first words at 2 years of age.

At the age of 8 years, the patient presented for evaluation following his first significant clinical episode. He experienced repeated yawning episodes while washing his hands, followed by loss of consciousness and loss of muscle tone which led him to fall to the floor. No accompanying shaking was observed.

On physical examination, the child weighed 23.5 kg (55th percentile), was 117 cm tall (21st percentile), and had a OFC of 57 cm (>97th percentile). Significant findings included macrocephaly, a broad forehead, a receding hairline with a low forehead attachment, narrow palpebral fissures, sparse distal eyebrows, a bulbous nasal tip, and small ears; he also had three hyperpigmented skin macules with jagged edges. The patient also had mild bilateral valgus foot and increased lumbar lordosis.

Neurological examination revealed normotonic and autonomous gait, dysmetria on cerebellar tests, poor motor coordination, and difficulty with fine motor tasks. Speech was poorly articulated, with phonological defects.

His overall intelligence quotient (IQ) was 84 (Wechsler Intelligence Scale for Children-WISC IV). No behavioral problems were reported or objectively assessed.

**Figure 1:**
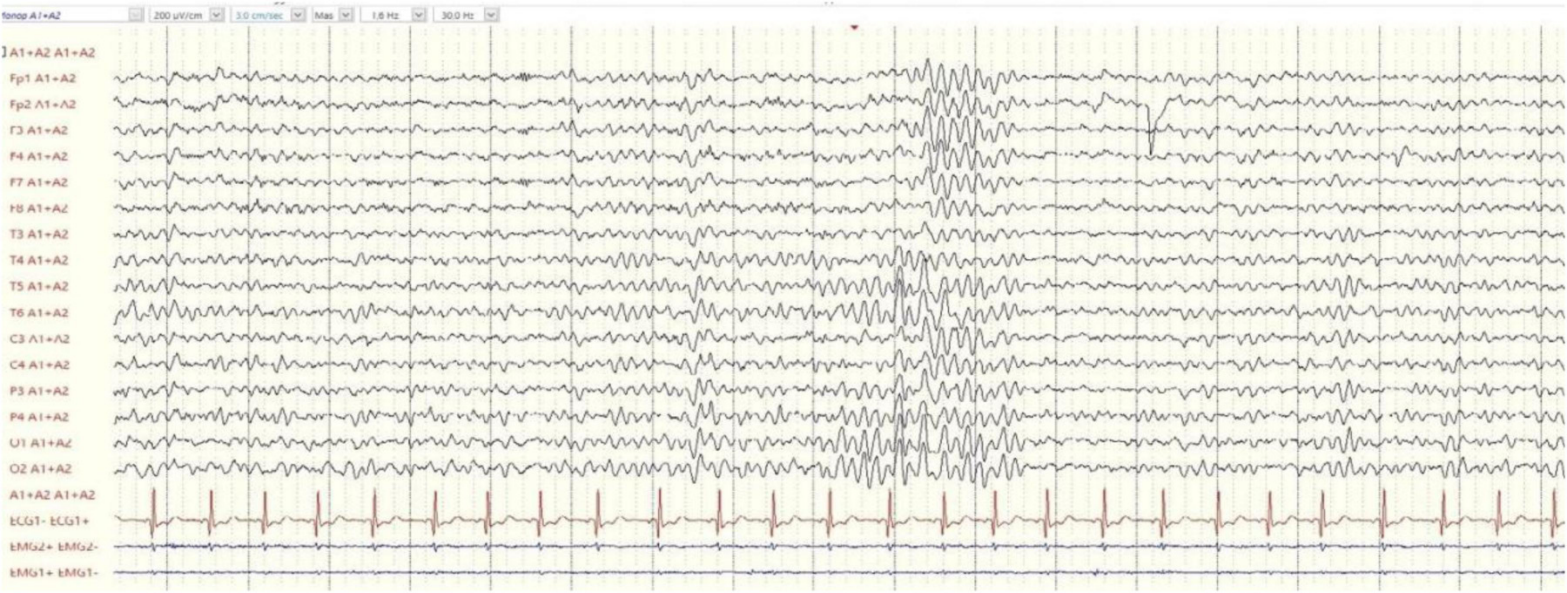
EEG at 8 years showing slowing of the posterior dominant rhythm and paroxysms of slow theta-band activity over the posterior regions.

Electroencephalography (EEG) revealed slowing of the posterior dominant rhythm and paroxysms of slow theta-band activity over the posterior regions (Figure 1).

**Figure 2:**
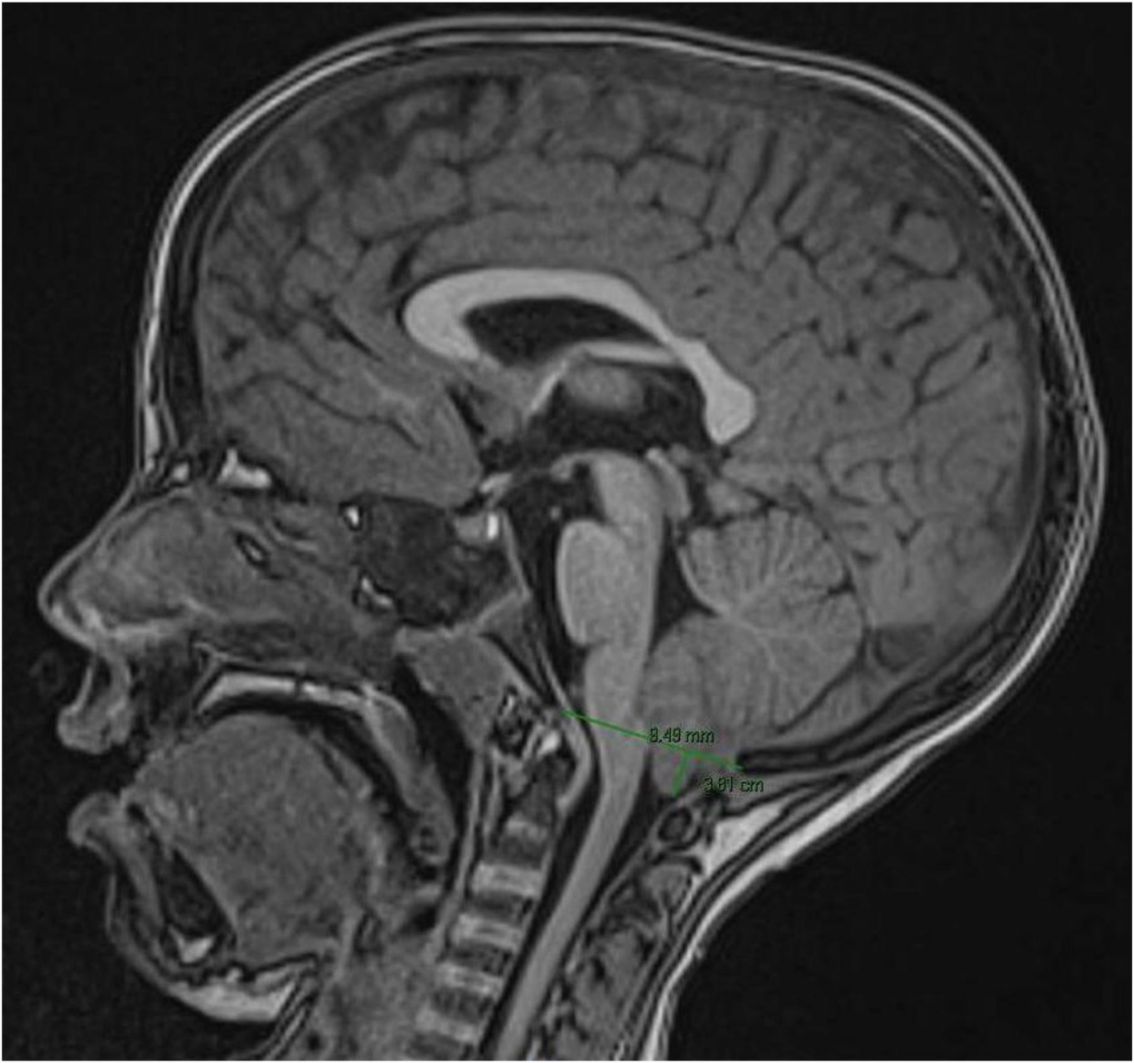
MRI at 8 years showing a type 1 Arnold-Chiari malformation, specifically a 9 mm herniation of the cerebellar tonsils beyond the foramen magnum.

Brain Magnetic resonance imaging (MRI) demonstrated a type 1 Arnold-Chiari malformation (CMI), specifically a 9 mm herniation of the cerebellar tonsils beyond the foramen magnum (Figure 2). The tonsils showed T1 hypointensity suggestive of early tissue suffering. No evidence of hydrocephalus or syringomyelia was detected. Despite the absence of syringomyelia or hydrocephalus, the patient was referred for neurosurgical evaluation because of the significant tonsillar herniation and the clinical history (in particular, the patient experienced an episode of loss of consciousness). Considering the combination of the clinical presentation and the neuroradiological findings suggestive of crowding at the craniocervical junction, the neurosurgical team recommended decompressive osteoligamentous surgery of the posterior cranial fossa with dural expansion.

Genetic analysis identified a novel missense variant, c.310G > A (p.Val104Met), located in exon 2 of the *NFIX* gene (NM_001365902.3), in a heterozygous state. This variant, never described before in the literature, arose *de novo* in the patient's DNA. Based on these results, the variant was classified as likely pathogenic and is considered responsible for the clinical presentation, compatible with MALNS.

During follow-up, additional evaluations revealed no abnormalities of the liver, spleen, kidneys or urinary tract, and no visual, fundus, hearing, or structural/arrhythmic cardiac defects.

## Diagnostic assessment

Genetic counseling was performed to evaluate the patient's personal and family history. Written informed consent was obtained from the parents at the Medical Genetics Unit of the Azienda Ospedaliero-Universitaria Senese for clinical exome sequencing, storage of clinical data, and the use of DNA samples for diagnostic and research purposes. Genomic DNA from the proband and both parents was extracted from peripheral blood samples collected in EDTA using standard procedures. Clinical exome sequencing was performed using a trio-based approach including the proband and both parents. Library preparation was performed using the Illumina DNA Prep with Enrichment kit with TruSight One Expanded oligonucleotide probes, targeting the coding regions of clinically relevant genes. Sequencing was carried out on an Illumina NovaSeq 6000 platform using massively parallel sequencing technology (Next Generation Sequencing).

Raw sequencing data were processed using Illumina BaseSpace Sequence Hub (Basic) for primary analysis, including read alignment to the human reference genome (GRCh37/hg19) and variant calling. Variant annotation and filtering were subsequently performed using the eVai software platform (enGenome, CE-IVD certified). Variants were prioritized based on allele frequency in population databases (e.g., gnomAD), predicted functional impact, inheritance model, and consistency with the patient's phenotype according to Human Phenotype Ontology (HPO) terms, including Global developmental delay (HP:0001263).

The analysis focused on a panel of genes associated with macrocephaly/overgrowth syndromes (*AKT1, AKT3, ASPA, ASXL2, BRWD3, CCND2, CDKN1C, CHD8, CUL4B, DHCR24, DIS3L2, DNMT3A, EED, EIF2B5, EZH2, GFAP, GLI3, GPC3, GPSM2, GRIA3, HEPACAM, HUWE1, KDM1A, KIAA0196, KIF7, KPTN, L1CAM, MED12, MLC1, MPDZ, NFIB, NFIX, NSD1, OFD1, PIGA, PIK3CA, PIK3R2, PTCH1, PTEN, RAB39B, RNF135, SETD2, SYN1, TMEM94, TSC1, TSC2, UPF3B,* and *ZBTB20*). No additional candidate variants explaining the clinical phenotype were identified. The mean coverage of the targeted regions was 121×, ensuring high analytical sensitivity for variant detection.

In parallel, cytogenetic analysis was performed on peripheral blood lymphocytes collected in heparin. Conventional karyotyping demonstrated a normal male karyotype (46,XY) in all analyzed metaphases.

The male proband was found to carry a heterozygous missense variant in exon 2 of the *NFIX* gene: c.310G > A (p.Val104Met). This variant results in the substitution of valine with methionine at codon 104 of the encoded protein. Trio analysis showed that the variant was absent in both parents, supporting a *de novo* occurrence in the proband. The presence of the variant in the proband and its absence in parental DNA were confirmed by Sanger sequencing.

The variant has not been previously reported in the literature or in major population databases. Variant pathogenicity classification followed the guidelines of the American College of Medical Genetics and Genomics (ACMG) and the Association for Molecular Pathology (AMP) for sequence variant interpretation, integrating population data, computational prediction tools, segregation analysis, and phenotype correlation. According to ACMG/AMP guidelines, the variant was classified as likely pathogenic, based on the following criteria: PS2 (*de novo* occurrence in the proband), PM2 (absent from population databases), PM1 (located in a mutational hotspot/domain), and PP3 (multiple in silico tools predict deleterious effect).

## Discussion

MALNS (OMIM #614753) is an overgrowth disorder caused by haploinsufficiency of the *NFIX* gene, resulting either from heterozygous chromosomal microdeletions involving the 19p13.2 region or from LoF variants in *NFIX*, which are almost exclusively located in exon 2 (5).

MALNS was first described in 2010 by Malan et al. (4). Subsequently, the phenotypic characteristics of this syndrome have been described by other Authors (3, 5, 8–18). Affected individuals typically have distinctive facial features, such as long and triangular face, prominent forehead with high anterior hairline, their nasal bridge might appear depressed, short nose with anteverted nares and upturned tip, deep-set eyes, down slanted palpebral fissures, long philtrum, small mouth that is often held open, thin vermilion of the upper lip, an everted lower lip, and a prominent chin. MALNS is characterized by a variety of clinical manifestations affecting multiple organ systems. One of the most consistent features in individuals with MALNS is developmental delay and intellectual disability, which has been reported in the vast majority of affected individuals, with severity ranging from mild to severe (18, 19). Seizures have also been described in individuals with MALNS. Cohort studies by Priolo et al. (5) and Macchiaiolo et al. (18) reported seizure frequencies ranging from approximately 13% to 26%. However, epileptic manifestations in MALNS remain incompletely characterized. A more recent caregiver-based survey including 53 individuals with MALNS reported seizures in approximately 47% of cases and EEG abnormalities in 55%, with a median age of seizure onset of around 3 years. In this cohort, drug-resistant epilepsy occurred in nearly one-third of affected individuals, and focal seizures or tonic–clonic seizures were the most commonly reported types (20). Additionally, autonomic symptoms such as episodic ataxia, dizziness, nausea, and postural fainting have been reported in some affected individuals. Neurobehavioral and psychiatric manifestations may also occur (6), including anxiety, mood instability, and hypersensitivity to noise. Skeletal involvement is commonly described and may include a slender body habitus and advanced bone age. Spinal abnormalities such as scoliosis or lordosis, as well as chest wall deformities including pectus carinatum and pectus excavatum, and pes planus have also been reported (18). Affected individuals may additionally present with long hands and fingers and an increased susceptibility to long bone fractures. Ocular involvement has been reported in several individuals, with refractive errors among the most frequently described findings. Other ophthalmologic features such as strabismus, nystagmus, blue sclerae, posterior polar cataracts, and optic nerve hypoplasia have also been described. Optic atrophy has also been reported in a patient initially diagnosed with SS who presented with progressive visual loss and was subsequently found to carry a pathogenic *NFIX* variant, confirming the diagnosis of MALNS (21). Cardiovascular abnormalities, including low-grade mitral regurgitation, aortic root dilation, and congenital heart defects, have been reported in some cases. Dental abnormalities such as malocclusion, dental caries, and oral apraxia have also been described. Hepatomegaly has been reported in occasional cases, while hearing loss and gastrointestinal issues such as constipation may occur in affected individuals (7).

CMI has been described too in association with MALNS (18, 22, 23).

CM are traditionally divided into four categories: types I through IV. Among these, CMI is the most frequently observed and is characterized by a downward slippage of the cerebellar tonsils for at least 5 mm into the spinal canal below the foramen magnum (24).

In 2010 Dolan et al. described a patient (patient 4) with a 19p13.13 microdeletion involving *NFIX* and CMI (22). Subsequently, two additional patients with *de novo* microdeletions on chromosome 19p13.2, associated with CMI were described by Shimojima et al. (23).

The study by Macchiaiolo et al. (2022) provided a comprehensive overview of the clinical features observed in patients with MALNS. A total of 16 patients with molecularly confirmed diagnoses were included; among them, 6 individuals (38%) were found to have a CMI. Of these 6 patients, one carried a 19p13.2 microdeletion, while the remaining five harbored pathogenic variants in the *NFIX* gene (18).

The exact mechanism responsible for the development of CMI in patients with MALNS is still unclear. However, some hypotheses regarding the functions of *NFIX* may provide an explanation. Indeed, NFIX is a 47 kDa dimeric DNA-binding protein that belongs to the Nuclear Factor 1 (NFI) family of transcription factors. NFI genes encode transcription factor proteins that contain a conserved DNA-binding and dimerization domain at the N-terminus and a transactivation/repression domain at the C-terminus. Members of the NFI family act as homodimers or heterodimers, binding to the palindromic consensus sequence TTGGC(N5)GCCAA with high affinity. Depending on the interactions with their dimerization partners, which may include other members of the NFI family, these factors act as dual transcriptional activators or repressors (4, 18).

*NFIX* shows strong expression in the central and peripheral nervous systems and in the perichondrium and plays a key role in the development of the brain - particularly the cerebrum and cerebellum - as well as in skeletal development and cartilage ossification (4). As demonstrated in animal models, in knockout mice *NFIX* deficiency leads to hydrocephalus, corpus callosum abnormalities, and severe skeletal defects, including delayed ossification and spinal malformations (25). These findings help us understand how disruptions in *NFIX* expression may contribute to structural anomalies like CMI in humans. Moreover, Tabata et al. (2020) described the case of a 5-year-old girl with a *de novo* insertion/deletion variant in exon 2 of *NFIX*, accompanied by hindbrain overcrowding. They hypothesized that, since the occipital bone develops through endochondral ossification, disruptions in this process could lead to underdevelopment of the posterior cranial fossa, resulting in hindbrain overcrowding. Additionally, megalencephaly may contribute to the narrowing of the posterior fossa (16).

No clinically significant genotype-phenotype correlations were found when comparing individuals with intragenic variants to those with gene deletions, except for a notably higher incidence of epilepsy in individuals with *NFIX* microdeletions (18). However, individuals with deletions encompassing *NFIX* and adjacent genes are more likely to experience seizures and EEG abnormalities compared to those with intragenic *NFIX* mutations. This difference may be due to the loss of the *CACNA1A* gene, located approximately 109 kilobases (kb) from *NFIX*. Apart from the increased prevalence of epilepsy, likely resulting from the deletion of *CACNA1A*, no significant phenotypic differences have been found between individuals with 19p13.2 microdeletions and those with intragenic mutations (5).

Given the rarity of MALNS, our case is particularly significant for several reasons. First, it adds a novel genetic variant to the existing body of knowledge, refining our understanding of the genetic heterogeneity of the condition. The identified variant is a *de novo* missense change affecting amino acid 104, located within the previously reported cluster of NFIX missense variants. It has not been reported in the GnomAD database. According to ACMG/AMP guidelines, the variant was classified as likely pathogenic. While most cases of MALNS have been associated with specific *NFIX* mutations, the identification of this novel variant in our patient further refines the clinical and molecular spectrum of the syndrome, which is essential for improving diagnostic accuracy and enabling more precise genetic counseling for affected families. Regarding neurological features, our case confirms the presence of CMI in MALNS. Although this finding has been previously reported, its documentation in our patient reinforces the phenotypic spectrum.

Overgrowth syndromes often share overlapping clinical features, particularly during early childhood. Therefore, access to early genetic testing is crucial for accurate diagnosis, especially in the presence of macrocephaly, developmental delay, and neurologic anomalies such as CMI. A timely diagnosis allows for personalized monitoring, appropriate interventions and family counseling.

Additionally, as MALNS is a multisystem disorder with a wide range of clinical manifestations, the identification of a novel *NFIX* variant in our patient contributes specifically to the refinement of genotype–phenotype correlations. The *de novo* missense variant p.Val104Met lies within the N-terminal DNA-binding/dimerization domain, in close proximity to a previously reported cluster of pathogenic variants, reinforcing the importance of this region for protein function. Although CMI has been reported in MALNS, its occurrence in our patient confirms the association of this neurological feature with variants in this domain, emphasizing the relevance of detailed phenotypic evaluation in conjunction with molecular data.

Our case underscores the need for clinicians to consider MALNS in the differential diagnosis, even in patients presenting with less common neurological features. The multisystem involvement highlights the importance of a multidisciplinary approach, including early genetic testing, targeted imaging, and appropriate interventions, to optimize clinical management and improve outcomes. Reporting novel *NFIX* variants is essential for refining allelic heterogeneity, supporting diagnostic recognition, and enhancing genotype-driven patient care.

## Conclusions

This case contributes to the growing clinical and molecular spectrum of MALNS by associating a previously unreported *NFIX* variant with CMI. It reinforces the importance of considering MALNS in the differential diagnosis of pediatric patients presenting with overgrowth, developmental delay, and posterior fossa anomalies. Early access to genetic testing plays a key role in reaching a timely diagnosis, differentiating between overlapping overgrowth syndromes, and guiding appropriate multidisciplinary follow-up. The report of novel variants is essential to deepen our understanding of genotype–phenotype correlations and to improve recognition and management of this rare condition.

## Data Availability

The original contributions presented in the study are included in the article/supplementary material, further inquiries can be directed to the corresponding author.
